# Human–Robot Intimacy: Acceptance of Robots as Intimate Companions

**DOI:** 10.3390/biomimetics9090566

**Published:** 2024-09-19

**Authors:** Sophia Bertoni, Christian Klaes, Artur Pilacinski

**Affiliations:** 1 CINEICC, University of Coimbra, 3000-802 Coimbra, Portugal; skbertoni@gmail.com; 2 Medical Faculty, Ruhr University Bochum, 44801 Bochum, Germany; christian.klaes@rub.de

**Keywords:** human–robot interaction, human–robot intimacy, intimate companion robots, sex robots

## Abstract

Depictions of robots as romantic partners for humans are frequent in popular culture. As robots become part of human society, they will gradually assume the role of partners for humans whenever necessary, as assistants, collaborators, or companions. Companion robots are supposed to provide social contact to those who would not have it otherwise. These companion robots are usually not designed to fulfill one of the most important human needs: the one for romantic and intimate contact. Human–robot intimacy remains a vastly unexplored territory. In this article, we review the state-of-the-art research in intimate robotics. We discuss major issues limiting the acceptance of robots as intimate partners, the public perception of robots in intimate roles, and the possible influence of cross-cultural differences in these domains. We also discuss the possible negative effects human–robot intimacy may have on human–human contact. Most importantly, we propose a new term “intimate companion robots” to reduce the negative connotations of the other terms that have been used so far and improve the social perception of research in this domain. With this article, we provide an outlook on prospects for the development of intimate companion robots, considering the specific context of their use.

## 1. Introduction

The idea of romantic relationships between robots and humans is often represented in popular culture. It was predicted by David Levy that, by 2050, robots will be immersed in society to such an extent that they could serve as romantic partners for humans and could even contract marriages with them [1]. When formulating this statement, Levy could not conceive of the COVID-19 pandemic, which sped up the process of the integration of robots into society. During that time, a higher demand for a specific type of robot arose: one that could fulfill the social and affective needs of individuals who were otherwise restricted [2]. These robots were called ‘companion robots’, i.e., social robots aimed at establishing closer relationships with users [2]. The interest in developing companion robots seems to be reflecting societal interest, driven by increasing risk of loneliness, as indicated by several reports [3,4].

In this paper, we summarize research on specific types of companion robots: those designed to provide intimate contact and sexual satisfaction to users. We outline the beneficial role that this type of robot can have while highlighting the risks they may carry for future human societies. Taking into account individuals’ varying levels of acceptance and reported cultural factors that might impact one’s established relationship with robots, we emphasize the importance of cultural context in forming bonds with robots, as it not only shapes these connections but also guides the direction of research and development. For instance, it has been proposed that in Western cultures (in this paper, mainly referring to the US and European countries), significant effort is still required to enhance robot acceptance [5]. In contrast, Eastern Asian societies exhibit a greater inclination toward integrating robots into service and healthcare sectors as previously reported [5]. This different level of acceptance extends to the development and release of robotic sex dolls [6], reflecting a cultural influence on the level of comfort with and openness towards robots for human intimacy.

## 2. Terminology

Companion robots are designed, among other functions, to address loneliness by providing social interaction [7]. Examples include robots such as NAO, Paro, and Pepper that were not designed with the primary goal of alleviating social isolation, but could also be used in this context [8,9,10]. However, these models typically fulfill only certain social needs, often overlooking a fundamental human need: intimacy, that is being close or bonded to someone else [11]. During the pandemic, the demand for yet another type of robot—sex robots [2]—increased significantly. These robots are specifically designed to meet users’ sexual needs, offering a form of intimacy that companion robots do not [12].

The terms ‘companion robots’ and ‘sex robots’ denote two distinct categories due to their inherent emotional component and each differs from other types of assistive robots, that is, robots aimed at generally assisting task execution [7]. This differentiation is evident from the prevalence of these terms in the research literature. A search on the Scopus platform reveals 2494 research papers on ‘assistive robots’ and 3381 on ‘collaborative robots’, compared to 636 papers on ‘companion robots’, 186 on ‘sex robots’, and only six on ‘intimate robots’. This disparity not only indicates a relatively low research interest in these specific types of robots but also suggests that companionship and sexual intimacy are regarded as separate domains, each requiring different robotic solutions.

The apparently low level of research interest in sex robots may be attributed to the novelty of the topic. However, it is also likely influenced by the general controversy surrounding these subjects, particularly in Western cultures, as we will demonstrate below. It is noteworthy that searching for ‘intimate robots’ did not return papers specifically about robots classified as intimate companions, but rather papers that mention robots and intimacy.

To start with, here we propose bringing together the concepts of companionship and sex as robot functions, under the term ‘intimate companion robots’ (ICRs), which can be used instead of ‘sex robots’. In our view, having a neutral term may facilitate the acceptability of ICRs due to having more acceptable cultural connotations. While more neutral than ‘sex robots’, we believe that the term “ICR” also carries a more complex meaning. This reflects the fact that, as companion robots become more complex, they may also perform a more diverse array of roles for their human users, from being a mere tool for simple tasks, to providing emotional support [12].

## 3. Perceived Benefits of ICRs

As ICRs are designed for providing a specific form of companionship, the main benefit of their use would be the mitigation of loneliness. Given that lack of intimacy is described as increasing the risk of loneliness [13], the available literature shows that there is more to this than a mere prediction.

A study conducted with elderly care home residents that interacted with Paro [14] demonstrated that interactions with companion robots can improve emotional well-being by decreasing loneliness. Additionally, this study found that residents not only experienced an enhanced emotional state after engaging with a companion robot but also increased their number of interactions with each other during sessions involving the robot.

This improvement is not only relevant when accounting for the aforementioned increased risk of loneliness, but also for populations more prone to isolation, such as the elderly, people with disabilities, and those with impaired social skills [6]. The positive impact on loneliness is, however, not the only identified benefit, as we will show below. 

For example, it is noteworthy that not only fully able, healthy people but also people with disabilities can benefit from using ICRs. As some researchers have pointed out, on one hand, people with disabilities face societal judgment, putting them in an almost asexual role, and, on the other hand, their disabilities may not allow them to explore and satisfy their sexual needs, both on a physical and a mental level [12]. In those cases, an ICR could serve as a body part aid, meaning it could support or perform the functions of an impaired or missing body part for physically impaired users [12]. ICRs may also provide intimacy, emotional support, and sexual companionship as a judgment-free tool, not only to disabled users, but also other individuals who may not want or do not feel capable of having a human partner to explore their sexuality [12,15,16] and fulfill their sexual desires [17]. On a cognitive level, people with disabilities may also benefit from the use of ICRs in the sphere of sexual education, learning about sexuality, emotions, and consent and preventing abuse [12,18]. ICRs may also support different therapies (psychological, physical, and sexual), being used both in assessments and interventions [16].

Other contexts in which the introduction of ICRs seems to be beneficial include the following: as a substitute for sex workers [1,6,18], in prisons [6], in contexts of social isolation [16,18], for the maintenance of a relationship [18], in research as a tool [16], and to satisfy transgressive sexual behaviors (those that are non-consensual or legally sanctioned). This latter use remains rather controversial, and considering the available data, the idea that ICRs could be used as a harm reduction tool by satisfying transgressive sexual needs has mixed empirical support [6]. One study found that individuals with pedophilic interests or a preference for non-consensual/violent sex were more open to engaging with ICRs compared to a control group [19]. However, another study revealed that sex offenders were not only less likely to engage with sex robots but also more skeptical about their potential to prevent sexual violence, compared to non-offenders [20]. This area of research is worth pursuing further due to its societal relevance and the significant risks posed by misconceptions regarding the use of ICRs for treating sex offenders.

## 4. Acceptance and Intimacy of ICRs

**Figure A1:**
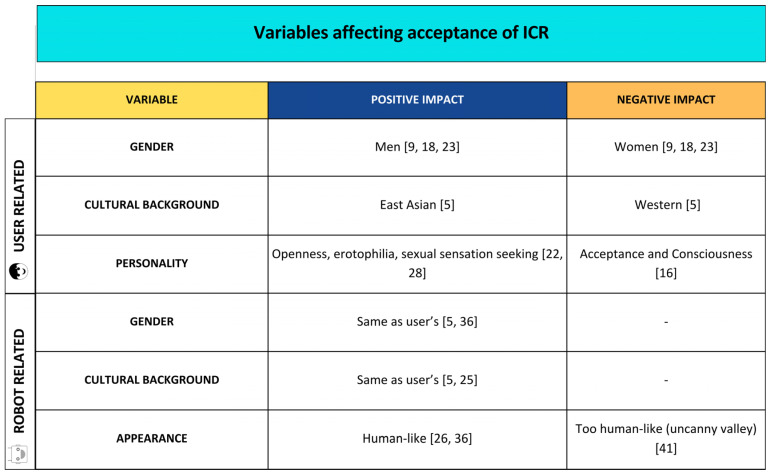
Identified variables that influence the acceptance of ICRs in a positive or negative way [5,9,18,22,23,25,26,28,36,41].

As mentioned earlier, the acceptance of ICRs depends on several factors, such as users’ cultural background, gender, etc., indicating a tangled network of relationships between these factors. In the following sections, we attempted to group and describe factors possibly important for the acceptance of ICRs, as indicated by the current research. These main factors are summarized in Figure A1 and discussed in detail.

When talking about ICRs and their scope of performed functions, promoting their acceptance is the first step to achieve intimacy. Intimacy with an ICR is limited to the feeling of bonding with it, and the literature suggests that intimacy is a predictor for positive sexual engagement, as pointed out by some authors [11,21].

### 4.1. Robot and User Characteristics

It appears obvious that the acceptance of ICRs relies on several factors related to both the user and the robot. These include the ICR’s appearance, the user’s characteristics, the robot’s features, the established interaction between the robot and the user, and culture. Given that ICRs (as a term and concept) are novel and not widely researched, the existing data refer to the acceptance of robots, sex robots, and companion robots, separately.

#### 4.1.1. Gender

Among user group characteristics, gender appears to be the main predictor for the acceptance of sex robots as listed by a number of authors [22]. Men tend to express more positive attitudes than women [9,18,22,23], and so do non-binary and gender nonconforming individuals. In their study, Nordmo et al. found gender has a significant effect on acceptance, with a significant interaction effect between gender and robot type (a sex robot or a platonic robot), with women showing significantly lower levels of acceptance of sex robots compared to platonic ones, while men showed a similar attitude towards both robot types [23]. It is noteworthy that the gendered difference in perceiving ICRs is likely unrelated to how different genders view sex with humans, even though heterosexual men find sex robots desirable for both personal use and sex work [6]. However, it is possible that those differences emerge from the fact that it is expected for men to express their interest in sex. This expectation is reflected in the current market, which obviously targets heterosexual men, with more women-like robots available [22]. This notion is further supported by data showing that women would express more interest in sex robots if they were specifically designed for them [24].

#### 4.1.2. Age

Across age groups, there is no clear trend in the acceptance of ICRs. For example, people 20 to 33 years old reported a lower level of acceptance of sex robots compared with older participants (ages 34 to 61) [18], but younger men (ages 20 to 30) were more accepting of robots in caring roles for the elderly (other factors included being educated and living in cities) [25]. This suggests an interaction effect between users’ gender and age on their acceptance of sex robots. Companion robots, on the other hand, seem to be more accepted across generations, with adults and children differing on the features they desire a robot to have [5]. One of these features is appearance: not only does the physical appearance of robots impact their acceptability, with more human-like robots being more accepted for intimacy [26], but also their gender. If the robot is of the same gender as the user, it is perceived as more acceptable [5]. However, the robot’s gender does not seem to impact the user’s trust in them, which is noteworthy, as trust is an important component for forming meaningful relationships [27].

#### 4.1.3. Personality

Among user characteristics, the role of personality seems to be elusive when it comes to predicting ICR acceptance. Although previous studies have focused on the effect of users’ Big Five traits on their acceptance of robots, concluding that Openness is a positive predictor and Acceptance and Consciousness are negative predictors [28], recent data show that, in addition to the Big Five traits, the users’ levels of erotophilia (willingness to engage in sexual activities) and sexual sensation seeking (seeking diverse types of stimuli) are better predictors, positively correlated with interest in and acceptance of sex robots [22]. In a study conducted with American men (who were particularly interested in elements of Japanese pop culture, like anime and manga) revealed that both shyness and self-isolation are positively related to accepting and purchasing a sex robot [29].

When it comes to sex robots, current data suggest that the feature that might enhance a robot’s acceptance the most is the quality of the sexual experience it provides, which plays a bigger role than its appearance [22]. For example, in an online study linking user personality traits and the acceptance of sex robots, it was determined that “erotophilic individuals seeking novel or more intense sexual experiences may be(come) the primary users of sex robots and influence their development”. This shows clearly that the acceptance of ICRs may primarily depend on users’ individual preferences and their readiness to accept an inanimate sexual partner, more than any other factor. As stated by the authors themselves, research on individualized preferences may be the most important when it comes to determining the acceptance of ICRs.

Apart from studying user personality traits, one can also ask about the perceived “personality” of ICRs. The influence of a robot’s personality, however, is still an open question, mainly because giving a personality to a robot is not as easy as giving it a gender or specific appearance [30]. From the available literature, it is possible to identify a few features that might enhance the acceptance of robots: matching a robot’s personality to the user’s expectations [30,31], being perceived as polite [32], displaying positive emotions (as long as it is not perceived as child-like) [33], having a playful personality [34], having feminine traits [30], and showing extroversion [31,35].

It is apparent that further exploration is necessary and that more personality-related traits should be analyzed and, perhaps, combined with motor characteristics, such as the robot’s specific patterns of movement and touch, in order to promote acceptance and intimacy.

### 4.2. Robotic Touch

Once the robot per se is accepted, we are confronted with the question of how tolerable an intimate relationship with it might be. One way of promoting intimacy is through touch [36], which has a number of sub-components influencing acceptance, such as the type of the touch, location of the touch, strength of the touch, social context, receiver’s expectation, and the robot’s body temperature [26,36,37]. A study with a Japanese sample, aimed at identifying what characteristics of touch would promote perceived intimacy between users and robots, suggested that patting increases the feeling of intimacy (compared to touching, stroking, and gripping), especially when using the fingers [36]. It seems that if the subjects do not want a relationship with the robot, they feel more intimacy through touches that feel less personal or that are not reserved for closer peers [36].

Another study suggested that, given participants’ reports, forming an emotional bond with a robot is perceived as less disturbing when compared to a physical bond formed through intimacy and sex [26], even with only 24.9% of the participants considering intimacy with robots possible. Then, when the participants had to touch the robot, touching areas considered less intimate (the head) evoked higher levels of electrodermal activity (indicating higher emotional arousal), in contrast to areas considered more intimate (the buttocks) [26]. While interesting, his finding can be alternatively explained by the fact that touching the head is usually more threatening than touching the buttocks, in terms of the potential physical damage that can be caused to the recipient. 

These findings suggest that intimacy and sexual contact with robots might be achieved by first establishing some sort of emotional relationship with them, in which the user would feel safe and comfortable. They also imply that methods for establishing emotional bonds with ICRs might be significantly moderated by cultural codes, such as the use of touch. We believe that cultural influences are not to be ignored when considering ICRs’ design and acceptance.

### 4.3. Cultural Influences on ICR Acceptability

Cultural context appears to play a fundamental role in the acceptability of ICRs, as it implies other factors identified as variables in the robot’s acceptance equation. For instance, the desired appearance and form of robots are different between cultures: while in the US, robots that imitate children, animals, or family members are considered inappropriate [18], in Japan, robots include childlike models that do not allow sexual intercourse (by not being penetrable) and “waifus” (fictional characters that one can consider their romantic partner) [6,38]. This indicates drastically different attitudes towards acceptable behaviors with robots between the US and Japan. Interestingly, the West has a lower level of acceptance of sex robots, but this has increased over time, with an observed 6% increase in Americans who would consider having sex with a robot between the years 2017 and 2021 (from 16% of the population to 22%) [12,22].

Culture also significantly influences people’s expectations of robot behavior. In Japan, robots are often viewed as independent beings with humanlike qualities [39], leading to a higher level of acceptance of humanoid forms [5,40]. In contrast, Western societies typically see robots as tools or aids [40], which can create a bias against their acceptance. Consequently, Americans report greater comfort with less humanoid robots, while Japanese individuals are more comfortable with humanlike robots [39]. Increasing the physical human likeness of a robot tends to cause more discomfort among Americans than Japanese people. Conversely, enhancing the emotional human likeness of a robot increases the level of comfort for Japanese individuals and decreases the level of comfort for Americans [39].

Culture might also be an important factor explaining the difference in robots’ presence in Eastern and Western societies, which is noteworthy since the world’s top three countries in robot use are in East Asia [40]. Not only are robots more present, but it appears that in East Asia, the population is generally receptive towards them [25]. As a result, this higher level of exposure to robots also shapes their acceptance, with a longer period of exposure over time generating a greater level of acceptance of robots, regardless of the user’s culture [25].

Studies on Eastern cultures have also considered a robot’s appearance and gender as factors influencing its acceptance [36]. The appearance of a robot is a complex issue in terms of acceptability. A robot can appear human-like only up to a certain point before it reaches the so-called ‘uncanny valley’. This phenomenon occurs when a robot’s attempt to closely mimic a living being falls short, resulting in feelings of repulsion rather than affection [41].

Taken together, these apparent cultural differences raise the question of why robots are more integrated into Eastern societies compared to Western ones. Interestingly, some authors suggest that this distinction is rooted in distinct cultural and religious heritage. In Eastern philosophies, such as Taoism, there is a belief in the unity between human and non-human beings (nature), which fosters greater levels of acceptance of robots. In contrast, Judeo-Christian traditions often emphasize a separation between humans and non-human entities, which may contribute to a lower level of acceptance of robots in Western societies [40]. To explore this distinction empirically, one study compared the gaze direction in pictures featuring both a robot and a human. Pictures were retrieved from search engines using the same keywords in English and in Japanese. In the English language context, the human and robot gazed at each other, in a so-called binary relationship. Meanwhile, in the Japanese language context, their gaze was directed somewhere else in a common direction, in a ternary relationship [40]. The Japanese context suggests that it might be that, in Japan, robots are treated as equals or at least as partners. This happens since the user and the robot show signs of joint attention, a preverbal form of communication based on directing or following the partner’s gaze to share an experience [42]. Therefore, the perception of a robot’s identity and human–robot relationships is influenced by cultural cues, which should be considered when exploring user acceptance of humanoid robots.

Culture not only influences the user’s perception of ICRs, but also shapes the way robots should be designed: the closer the robot is to the user’s culture or ethnicity, the more the user anthropomorphizes it [5]. It follows that anthropomorphization might enhance social connections between humans and robots. In addition, when robots behave according to the user’s culture or speak in the user’s native language, they are more likely to be perceived as a group member [25]. Therefore, we need to consider how important it is that a robot matches the user’s cultural backgrounds in order to achieve a higher level of acceptance of ICRs.

However appealing, the idea of assuming that robots are universally accepted in Eastern societies may not be completely true. In some studies, Japanese populations have not shown this putative higher level of acceptance, reporting feeling less safe around robots compared to French populations [25]. They also did not show a higher level of comfort around robots compared to Americans in one study [39] and presented less positive results than other cultures for the Negative Attitudes Towards Robots Scale in another [25]. This could be an effect of the aforementioned higher level of exposure, as having more contact with robots, the Japanese may have a greater understanding about the advantages and disadvantages of robots and might have a more realistic view on human–robot interactions (HRIs) [25]. It might also be that, despite having a lower level of exposure to robots and having less experience with HRIs, the Western population ends up with a more positive attitude towards robots after interacting with them than the Eastern population [25].

Likewise, it is important to note that dividing cultural differences into Western and Eastern categories can be misleading, as these are broad generalizations, and significant variations can exist within each group. For example, Italian and British participants might perceive interactions with robots differently to each other, with the latter reviewing them in a more negative way [5]. In another case, the Japanese perspective can be closer to the American perspective than to the Korean perspective in terms of the level of skepticism about the employment of robots [25].

Therefore, it is conceivable that culture is a strong modeling factor for the acceptance of ICRs, directly and indirectly, by shaping the other relevant factors identified here to provide higher levels of acceptability. In any case, cross-cultural studies on the acceptability of ICRs need to avoid oversimplification while accounting for cultural idiosyncrasies. 

## 5. Possible Caveats of ICRs

**Figure A2:**
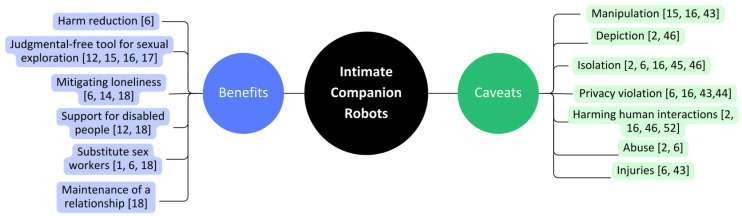
An overview on ICR-related effects on society, in terms of ICRs’ possible benefits and caveats identified by the previous literature [1,2,6,12,14,15,16,17,18,43,44,45,46,52].

Though ICRs appear to present a viable opportunity to alleviate loneliness and satisfy intimate needs, due to the machine-like nature of robots, there are several risks concerning their use, as outlined by multiple authors. We will now discuss these caveats, which we summarize in Figure A2.

### 5.1. Technological Implications

Given that a robot is fundamentally a human-designed machine, it is prone to malfunctions that can lead to harm to users, such as injuries resulting from sex toys, on a physical, biological, and chemical level [6,43]. Additionally, a robot may also be vulnerable to security breaches, risking the exposure of the user’s intimate data [6]. Moreover, the user’s intimate data are inherently at risk, as some ICRs are equipped with cameras or connected to artificial intelligence systems, making user privacy questionable [44]. The protection of such intimate data is not yet regulated due to the specific nature of ICRs, leaving users vulnerable to data collection, either officially or through data leaks [43]. Users are also vulnerable to blackmailing by those who might have access to such data and who might attempt to influence or manipulate the robot in possession of the user’s intimate data [16,43].

### 5.2. Psychological and Behavioral Implications of ICR Use

A common concern in the literature is the possible negative impact of HRIs with companion or sex robots on human–human interactions. Though robots aim to mitigate loneliness, they might have a paradoxical effect of reinforcing this loneliness by satisfying users’ needs and leading them to not seek for human companions anymore, to isolate themselves from society, or to have worsened social skills [2,6,16,45,46]. Targeting isolated or lonely people, ICRs can also turn those people into commercial targets, with their vulnerability being exploited for the profit of the manufacturer, turning loneliness into a marketing strategy [47].

Human–robot intimacy can also reinforce unhealthy mental states by fostering deception. Users may project onto the robot human traits that it does not possess, leading to a dissociation between the objective reality and the subjective experience of the interaction [2,46]. This deception can also give users a false understanding of human interactions. It can also promote obsessive-compulsive behaviors or behaviors related to addiction in the user [16].

Due to its likely passive behavior, the robot will not reject or confront the user but will instead reinforce and meet their expectations, responding positively to the uservs behavior [2,46]. This can result in decreased tolerance for relational frustrations, which are unavoidable in interactions with other humans [2]. It can give a false impression of sexual availability and grant sexual experiences that would not likely occur in reality [16].

The passive position of the robot leaves it vulnerable to abuse from the user’s side, say by violent behavior, both in sexual and non-sexual contexts [2,6]. Using robots to satisfy sexually violent desires may not be sustainable in the long term. Since robots cannot give or deny consent, these interactions are not truly non-consensual, which may reduce user satisfaction over time. Consequently, users might eventually seek out non-consensual contact with humans [6].

Moreover, since ICR robots often possess female physical features, probably due to the perceived gendered role they perform [48], and exhibit the stereotypical behaviors of women (e.g., serving men), interacting with such robots may foster negative attitudes towards human women, as suggested by some authors [2,6,16,17]. Interestingly, research fails to account how male-looking sex robots might inflict the same damage on human men [49].

Including a robot in an existing relationship, such as using it as a sex partner, can raise concerns about increasing tensions between partners due to jealousy [23,46] or distorted expectations of human interactions. As jealousy may arise from perceived infidelity [50], it constitutes yet another harm, as infidelity is known for being harmful to monogamous relationships [51]. Participants had lower assessed levels of perceived infidelity when considering their partner being with a sex robot than when considering them being with another human partner. However, if the participants were not told whether their partner was with a sex robot or with a human partner, 52men showed lower rates of perceived infidelity [52].

Additionally, it is important to consider that robots could be misused by perpetrators of domestic abuse, either as a tool to facilitate abuse against their partner or as objects of abuse themselves [53].

Lastly, when used in care-taking services, as much as ICRs might provide people with disabilities with companionship and allow them to explore their sexuality, the type of disability should be considered. People with cognitive impairments may struggle to distinguish a humanoid robot from a human or may not fully understand or appreciate their actions, potentially rendering that population incapable of giving informed consent [17]. This vulnerability puts them at risk of sexual, emotional, or psychological exploitation and abuse, as robots might not respect their boundaries, for example.

In sum, ICRs can be a viable alternative for people deprived of social contact. It is necessary to treat relationships established with robots with caution, as robots’ non-human nature may lead to their misuse and may cause users to be less tolerant of human behaviors and interactions. As discussed above, research to-date shows that the most critical risk from the use of ICRs is to humans: not only to ICR users but also to their social environment. 

## 6. Conclusions

Forming bonds with robots is an alternative approach to current issues related to loneliness and isolation that some groups face. It can also be an aid for spreading awareness about sexual education, abuse, and violent sexual behaviors, or for replacing sex workers who might not be protected by law.

It is thus important to understand how to best foster this connection and how to make it safe and ethical. For that, more research is needed to better understand how HRIs can impact and alter human behavior and human relations. Establishing an ethical framework for HRIs is also necessary to prevent the abuse of vulnerable groups as well as to avoid abuse towards robots, which could later be translated into abuse towards human peers.

We analyzed factors that could interfere with the bonding between humans and robots, through the levels of acceptance of humanoid robots and intimacy with them. The literature suggests that cultural context is crucial in HRIs. It not only influences the type of interaction and level of acceptance but also shapes other factors that enhance HRIs, such as exposure to robots, expectations of robot behavior, and preferred robot appearance. Moreover, the closer the cultural context is between the user and the robot, more likely an ICR is accepted.

There is still a need for more research in terms of different cultural contexts and levels of robot acceptance, accounting for distinctions within traditional cultural clusters (for example, Western and Eastern) and individual differences (e.g., personality traits and experience). Understanding individual differences is also crucial and yet has not been explored very much, calling for further investigations into this topic. Research on ICRs should also delve into African and South-American cultures, which comprise Western societies as well.

Future studies on ICRs should also account for the influence of robot acceptance factors that come from other domains of social robotics (shown in Figure A1), as these can lead to biases in the results. For example, cross-cultural studies should consider not only the participants’ cultural background, but also their gender and personality, as well as those of the robot.

We believe that research on ICRs, regardless of cultural taboos, is needed in order to mitigate future risks coming with the unavoidable introduction of robots into all, even the most intimate, spheres of human life.

## Data Availability

Not applicable.
